# A New Phenotype of Ataxia With Oculomotor Apraxia Type 4

**DOI:** 10.7759/cureus.13601

**Published:** 2021-02-28

**Authors:** Eduardo Freitas, Octávia Costa, Sofia Rocha

**Affiliations:** 1 Neurology, Hospital de Braga, Braga, PRT; 2 Neurology, Hospital de Viana do Castelo, Viana do Castelo, PRT

**Keywords:** ataxia, oculomotor apraxia, epilepsy, dystonia, gait, cerebellar atrophy

## Abstract

Ataxia with oculomotor apraxia is a rare neurodegenerative subgroup of diseases with manifestations that include cerebellar ataxia, oculomotor apraxia, extrapyramidal features, and sensorimotor neuropathy. In 2015, ataxia with oculomotor apraxia type 4 was described in 11 Portuguese individuals. The mean age of onset was 4.3 years, with severe extrapyramidal manifestations, neuropathy, rapid progression, and ataxia, being wheelchair-bound during adolescence. The disease is caused by homozygous or compound heterozygous mutations in the PNKP gene. In this case report, we describe two sisters, who were 52- and 58-years-old, with cerebellar dysarthria, oculomotor apraxia, dystonia, and gait ataxia. Two new mutations in the PNKP gene were detected in both sisters, confirming the diagnosis of ataxia with oculomotor apraxia. They were remarkable because they were able to walk unaided during adulthood and had epilepsy. With these clinical cases, we attempt to raise awareness of the possibility of different phenotypes of this rare disease, expanding the spectrum of manifestations of ataxia with oculomotor apraxia type 4.

## Introduction

Ataxia with oculomotor apraxia (AOA) is a subgroup of hereditary autosomal recessive (AR) cerebellar ataxias involving cerebellar ataxia, sensorimotor axonal neuropathy, oculomotor apraxia, and extrapyramidal features. AOA type 4 (AOA4) was described in 2015 in a Portuguese population of 11 patients with early-onset ataxia who were found to have mutations in the PNKP gene and were homozygous or compound heterozygotes for these mutations [1]. After Friedreich ataxia, AOA4 is the most frequent AR ataxia in the Portuguese population following a population-based nationwide survey performed between 1994 and 2004 [1]. The mean age of onset was 4.3 years old, with marked extrapyramidal manifestations, rapid progression, metabolic changes with elevated alfa-fetoprotein and hypercholesterolemia, severe neuropathy, and ataxia with all the patients being wheelchair-bound during young adulthood. None of the patients had epilepsy [1-2]. We describe two sisters with two new PNKP mutations, which, besides the typical clinical manifestations of AOA4, are unique because they had epilepsy and remained fully ambulatory until late adulthood.

## Case presentation

Patient 1

**Figure 1:**
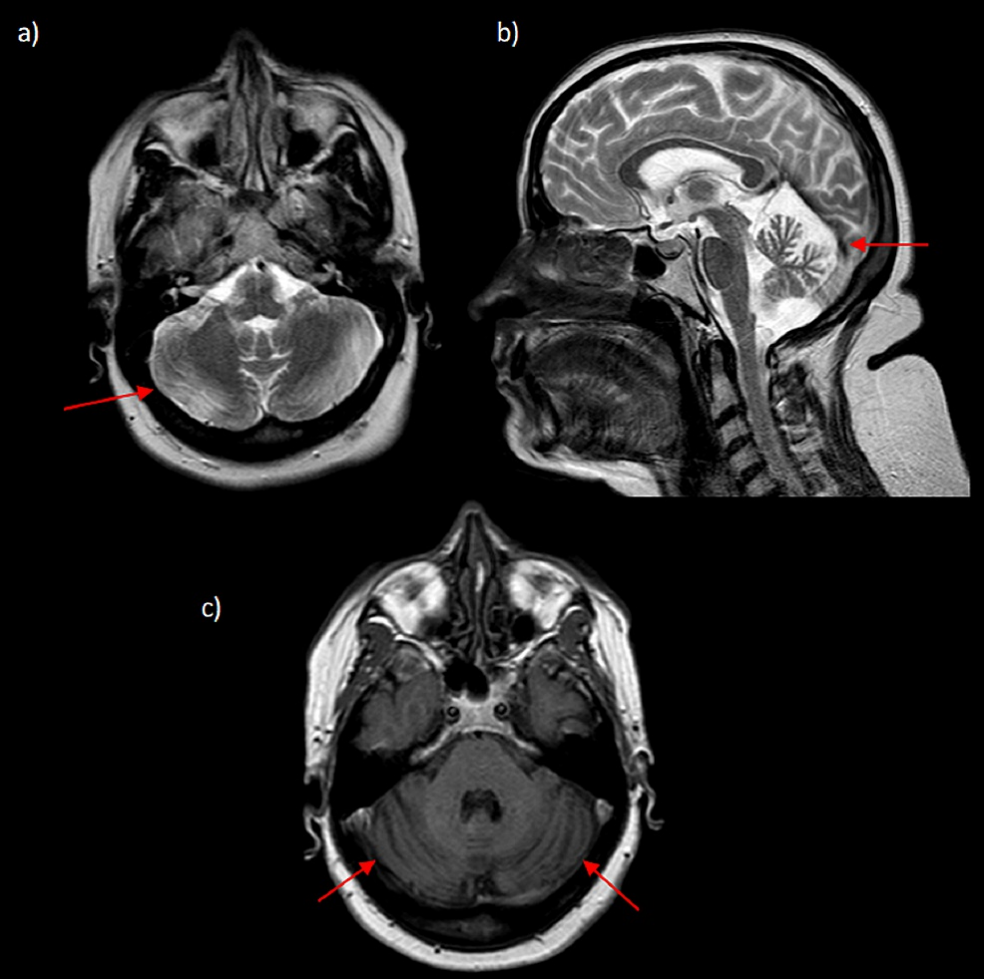
Brain MRI of Patient 1 a) Axial T2 sequence showing cerebellar atrophy; b) Sagittal T2 sequence showing cerebellar atrophy and microcephaly; c) axial T1 sequence with evident cerebellar atrophy

A 52-year-old female was admitted to the emergency room due to a focal status epilepticus. Reviewing the clinical history, the patient was able to complete high school and work in a factory until the age of 45 despite having difficulties walking since early childhood, and the family reported that she seemed to have gaze direction difficulties. Family history showed seven siblings, three of which also had difficulties with balance (two females and one male). On neurological examination, we noted microcephalus, obesity, cervical dystonia, oculomotor apraxia, cerebellar dysarthria, and gait ataxia with important balance impairment but still able to walk without support. She had no changes in sensory testing and osteotendinous reflexes were present. The blood tests showed elevated alfa-fetoprotein and elevated cholesterol, the electroencephalogram (EEG) showed a 7 Hz, posterior, symmetrical, reactive basal activity without clinical or electrical seizures, and the brain magnetic resonance imaging (MRI) showed cerebellar atrophy without lesions of the parenchyma on T2-weighted fluid-attenuated inversion recovery (T2/FLAIR) or T1 sequences (Figure 1).

The patient refused electromyography but there were no clinical signs of polyneuropathy. She was medicated with levetiracetam (3 grams/day) and topiramate (200 mg/day) without seizure recurrence. Genetic testing with next-generation sequencing (NGS) for hereditary ataxia was performed and two compound heterozygous mutations in the PNKP gene were found (c.1029+2T>C and c.1221_1223del) compatible with the diagnosis of AOA4.

Patient 2

A 58-year-old female with a previous history of epilepsy since childhood, hypercholesterolemia, and obesity was medicated with phenobarbital 100 mg/day and atorvastatin 10 mg/day. She worked in a factory until the age of 45. She was referred to the outpatient clinic because of dysarthria and gait balance impairment. She was the older sister of Patient 1. The neurological examination was remarkable and showed moderate cerebellar dysarthria, fragmentation of the ocular movements in smooth pursuit, oculomotor apraxia, superior right limb dystonia, and gait balance impairment, with impossible tandem but able to walk unaided. She had no sensory changes and osteotendinous reflexes were normal. The blood tests showed elevated alfa-fetoprotein and cholesterol, the EEG did not show epileptic activity. A neuropsychological evaluation was performed and showed multidomain mild cognitive impairment. The patient refused the brain MRI or CT and electromyography but there were no signs of polyneuropathy in the neurological examination. Genetic testing with the Sanger method was performed and two compound heterozygous mutations in the PNKP gene were found (c.1029+2T>C and c.1221_1223del), confirming the diagnosis of AOA4. The remaining siblings were referred to genetic counseling but refused to be tested.

## Discussion

AOA4 is a rare neurodegenerative disorder recently described in a Portuguese population. It is caused by mutations in PNKP. This gene has important roles in multiple pathways involved in deoxyribonucleic acid (DNA) damage repair, including single-strand breaks (SSBs) and double-strand breaks (DSB) [3]. It was originally associated with early infantile epileptic encephalopathy 10, which is characterized by microcephaly, seizures, and developmental delay [1]. It was also associated with cerebellar atrophy in two siblings with progressive polyneuropathy, microcephaly, intellectual disability, and mild epilepsy without oculomotor apraxia [4]. The different phenotypes so far associated with mutations in PNKP do not seem to relate to either the type or the location of the mutation, and they may reflect a gene-environment interaction [1].

AOA4 is the second most frequent cause for recessive ataxia in Portugal, after Friedreich ataxia, and the most frequent form of AOA. Most of the described patients had dystonia as the first manifestation, followed by ataxia and oculomotor apraxia. Most individuals had signs of polyneuropathy with generalized areflexia. Loss of ability to walk occurs seven to 21 years after disease onset, with the individuals being wheelchair-bound during adolescence. Cognitive impairment is frequent. Alfa-fetoprotein and cholesterol are usually elevated. Brain MRI showed cerebellar atrophy in all patients [1].

## Conclusions

We present the clinical cases of two sisters with a new phenotype of AOA4, and two new PNKP mutations, which have some typical features of the disease but without relevant polyneuropathy and with epilepsy and the ability to walk unaided during adulthood. The clinical picture of the two sisters with AOA4 is unusual, and to our knowledge, there are no reports of patients with these features, thus expanding the phenotype of this rare disease.
